# Immunostimulatory effects of three classes of CpG oligodeoxynucleotides on PBMC from HCV chronic carriers

**DOI:** 10.1186/1476-8518-6-3

**Published:** 2008-06-09

**Authors:** Curtis L Cooper, Navneet K Ahluwalia, Susan M Efler, Jörg Vollmer, Arthur M Krieg, Heather L Davis

**Affiliations:** 1 Division of Infectious Diseases, University of Ottawa at The Ottawa Hospital and Ottawa Health Research Institute, Ottawa, Canada; 2 Coley Pharmaceutical Canada, Ottawa, Canada; 3 Coley Pharmaceutical GmbH, Langenfeld, Germany; 4 Coley Pharmaceutical Group, Wellesley MA, USA

## Abstract

**Background:**

Chronic hepatitis C virus (HCV) infection results from weak or absent T cell responses. Pegylated-interferon-alpha (IFN-α) and ribavirin, the standard of care for chronic HCV, have numerous immune effects but are not potent T cell activators. A potent immune activator such as TLR9 agonist CpG oligodeoxynucleotide (CpG) may complement current treatment approaches.

**Methods:**

Peripheral blood mononuclear cells (PBMC) obtained from HCV chronic carriers who failed previous treatment and from healthy donors were incubated *in vitro *with the three main CpG classes (A, B or C), recombinant IFN-α-2b (IntronA) and/or ribavirin. Proliferation and cytokine secretion (IFN-α, IL-10 and IP-10) were evaluated.

**Results:**

CpG induced proliferation and cytokine secretion in patterns expected for each CpG class with similar group means for HCV and healthy donors. IntronA and ribavirin, alone or together, had no detectable effects. IntronA and C-Class CpG together induced more IFN-α than CpG alone in most subjects. IFN-α secretion was proportional to the number of plasmacytoid dendritic cells in PBMC from healthy donors but not HCV donors in whom responses were highly heterogeneous.

**Conclusion:**

The strong immune stimulatory effect of CpG on PBMC isolated from treatment-failed HCV patients suggests possible utility alone or in combination with current HCV antiviral treatment.

## Background

Hepatitis C virus (HCV)-induced liver disease is an important health issue [1,2]. Acute infection usually is not spontaneously cleared in part due to immune escape by emerging quasispecies [3] and virus-induced immune dysfunction. HCV-specific Th1-type immune responses, which are considered essential for longterm viral control and eradication [4,5] are stronger and broader in those with self-resolving acute infection in comparison to those who go on to develop chronic disease [6-9]. These responses improve during therapy but remain much weaker than with self-resolving infection [10-12]. This suggests that the relatively poor response (< 50% for genotype 1) achieved with pegylated-interferon-alpha (PEG-IFN-α) and ribavirin[13] may be due to inadequate immune stimulation. PEG-IFN-α and ribavirin both appear to possess anti-viral and some immune modulatory activities [14,15]. Although the mechanism of ribavirin activity remains unresolved this medication may enhance virological and biochemical responses that are associated with faster second phase viral decay with consequent accelerated reduction in the pool of infected cells [16-19]. Ribavirin activity may be mediated by reduced T cell production of IL-10 [20-22]. IL-10 has been proposed to promote the formation of regulatory T cells (Treg) in chronic HCV that inhibit the generation of desirable Th1 type T cell responses [23]. However, neither PEG-IFN-α nor ribavirin appear to be a potent immune stimulators [24,25]. As such, HCV treatments may benefit from more potent immune modulators used alone or in combination with current treatment regimes.

Toll-like receptors (TLR) expressed by immune cells recognize specific pathogen-associated patterns, and play a critical role in regulating innate and adaptive immunity [26,27]. Synthetic oligodeoxynucleotides (ODN) containing immunostimulatory CpG motifs (CpG) directly activate human B cells and plasmacytoid dendritic cells (pDC) through TLR9 [28]. Other immune cells are indirectly activated. CpG has potential utility in HCV via multiple mechanisms of viral control. These include activation of natural killer (NK) cells which clear virus from infected hepatocytes during acute infection [29-31], pDC maturation for improved antigen presentation, and enhanced Th1 cytokine profiles (IL-12, IFN-γ and many IFN-α subtypes) that have known antiviral properties and promote Th1-biased lytic and non-lytic T cell responses [32]. This former property is observed even in the presence of pre-existing Th2 responses [33].

CpG properties vary depending on length, sequence, backbone and formation of secondary or tertiary structures. Three main classes of stimulatory CpG are described [34]. A-Class CpG is synthesized with a chimeric backbone with nuclease resistant phosphorothioate 5' and 3' ends and a native phosphodiester central CpG motif region. These molecules form higher ordered structures and are characterized by strong NK cell and pDC activation, high levels of IFN-α production, and limited B cell activation [35-38]. B-Class CpG are phosphorothioate throughout and do not form secondary structures. They are characterized by strong B cell activation [39], moderate NK activation [29], and pDC activation with moderate IL-12 and limited IFN-α production. C-Class CpG are phosphorothioate molecules with a 3' palindrome region that permits stem-loops and duplexes. They have properties intermediate to A- and B-Classes with good B cells and NK cells activation, and induce DC IFN-α secretion [38,40,41]. The higher order structures of A- and C-Classes appear to affect intracellular localization and facilitate cross-linking of TLR9 receptors, which may be associated with IFN-α induction.

A B-Class CpG has entered clinical testing and has demonstrated efficacy together with doublet chemotherapy in a Phase II study in non-small cell lung cancer [manuscript submitted] and as a hepatitis B vaccine adjuvant [42] in healthy volunteers [43,44] and vaccine hyporesponsive HIV-infected patients [45]. Based on this knowledge, we evaluate the ability of different CpG classes to stimulate immune cells from healthy or HCV-infected donors to proliferate and secrete key cytokines.

## Methods

### Human PBMC

Peripheral blood mononuclear cells (PBMC) were recovered from 27 adult volunteers (12 healthy, 15 HCV treatment refractory) at The Ottawa Hospital, Ottawa, Canada under informed consent and IRB approval. Subjects with other chronic infections or who had received HCV therapy within 3 months were excluded. Viral genotypes for the 15 HCV-infected subjects was: 1b (n = 6), 1a (n = 5), 3a (n = 3) and 4c (n = 1). PBMC were purified from whole blood (200 ml, venous puncture, heparinized vacutainers) by centrifugation over Ficoll-Pacque (Amersham Pharmacia Biotech, Uppsala, Sweden) at 400 × g for 35 min. Cells were resuspended in RPMI complete media containing 10% normal human AB serum (heat inactivated) and 1% penicillin/streptomycin at 10 × 10^6^/ml and used fresh to assay cytokine secretion and proliferation.

### Reagents

ODN sequences were: A-Class CpG (2336; GGG*G*A*C*G*A*C*G*T*C*G*T*C*GGGGGG), B-Class CpG (2006; TCGTCGTTTTGTCGTTTTGTCGTT), C-Class CpG (2429; TCGTCGTTTTCGGCGGCCGCCG) and non-CpG control (4010 ; TGCTGCTTTTTGCTGGCTTTTT). B- and C-Class CpG had entire nuclease resistant phosphorothioate backbones. A-Class CpG had chimeric backbone with central phosphodiester region (indicated by *) and phosphorothioate ends. All ODN, verified to be endotoxin-free (Coley Pharmaceutical GmbH; Langenfeld, Germany), were resuspended in TE buffer at pH 8.0 (OmniPur^®^; EM Science, Gibbstown, USA) and diluted in RPMI 1640 complete media (GibcoBRL, Grand Island, USA) containing 10% heat inactivated, normal human AB serum (Wisent, St. Bruno, Canada) and 1% penicillin/streptomycin (GibcoBRL) just prior to use in cell assays.

Phytohemagglutinin (Sigma-Aldrich, Oakville, Canada), positive control in cell stimulation assays, was diluted in media then added to cells for final concentration of 10 μg/ml.

IntronA (Schering, Pointe-Claire, Canada) was added to the culture media for final concentrations of 125 or 1000 IU/ml. Ribavirin (CN Biosciences, La Jolla, USA) was reconstituted to 500 μM with sterile distilled water then diluted in media and added to cells for final concentration of 5 μM.

### Immune assays

#### Cytokine Assays

Freshly isolated PBMC (1 × 10^6 ^in 200 μl complete RPMI media) were incubated at 37°C with 5% CO_2 _in 96-well flat-bottom plates with ODN at 3 or 6 μg/ml (approximately 0.5 and 1 μM). Cell supernatants collected after 48 hrs were stored at -80°C until assayed. Media alone and PHA were negative and positive controls respectively.

Commercial ELISA kits were used according to manufacturer instructions to measure IP-10, IL-10 (R&D Systems, Minneapolis, USA) and multi-species human-IFN-α (PBL Biomedical Laboratories, Piscataway, USA). The kit specified detection limits were used for ELISA values below these limits (16, 23 and 31 pg/ml for IP-10, IL-10 and IFN-α respectively).

Preliminary dose-response data for CpG on PBMC from 3 healthy donors cultured with C-Class (1, 3, 6, 9 and 12 μg/ml final concentration) and B-Class (1, 3, and 6 μg/ml) CpG showed maximum responses 3 μg/ml for IFN-α and at 6 μg/ml (B-Class) or 12 μg/ml (C-Class) for IP-10 and BCP levels. Due to blood volume limitations, CpG was tested only at 3 and 6 μg/ml for B- and C-Classes (approximately 0.5 and 1 μM respectively) and 6 μg/ml for the A-Class.

### PBMC proliferation

ODN solutions (100 μl) were added to 96 well plates to give final concentrations of 3 or 6 μg/ml. Isolated PBMC were resuspended at 1 × 10^6^/ml in complete RPMI media and 100 μl of cells were added to each well and cultured for 5 days at 37°C with 5% CO_2_. Cells were pulsed with ^3^H-thymidine (1 μCi/well) for 18 h then harvested onto filter paper; radioactivity was measured and reported as a stimulation index (SI) relative to untreated media control.

### Identification of pDC by flow cytometry

Three-colour immunofluorescent flow cytometric analysis was used to quantify pDC. 3 × 10^6 ^PBMC were resuspended in 300 μl of complete RPMI media and divided among three tubes, one as negative control (autofluorescence), and two for pDC detection of lineage negative, CD11c negative, HLA-DR positive, and either BDCA-4 positive or CD123 positive. Monoclonal antibodies were: Mouse IgG1 Anti-Human BDCA-4-PE (Miltenyi Biotech, Auburn, USA), Mouse IgG1 Anti-Human CD123-PE (BD Biosciences-Pharmingen, San Diego, USA). Mouse Anti-Human CD11c-PC5 (BeckmanCoulter, Fullerton, USA), Mouse IgG1 Anti-Human HLA-DR-ECD (BeckmanCoulter) and a FITC-conjugated mouse IgG1, IgG2b anti-human lineage cocktail including CD3, CD14, CD16, CD19, CD20, CD56 (BD Biosciences-Pharmingen). Staining was per manufacturer recommendations; analysis by flow cytometry counted 50,000 events per sample (Beckman Coulter Epics XL-MCL, Expo32 software).

### Epstein Barr Virus immortalized B-cell lines

Healthy PBMC from 5 donors were resuspended in 2.5 ml of RPMI media (5 × 10^6 ^cells) containing 10% fetal bovine serum (GibcoBRL) and 1% penicillin/streptomycin. Epstein-Barr virus (EBV)-containing supernatant (2.5 ml) previously collected from a EBV transformed B cell line (B95-8, ATCC, Manassas, USA) per manufacturer instructions was mixed with PBMC and incubated 2 hr at 37°C with 5% CO_2_. Cyclosporin A (Sigma-Aldrich) at 1 μg/ml in RPMI complete media was added to a final volume of 10 ml and cells were grown 4 wk in flasks at 37°C with 5% CO_2_.

### Statistical analysis

Data were expressed as group means ± standard errors of the means (SEM) for absolute data. Student's *t *test was used to compare two groups and one-factor analysis of variance (ANOVA) followed by the Mann Whitney Test for three groups or more.

## Results

### Cytokine secretion

**Figure 1:**
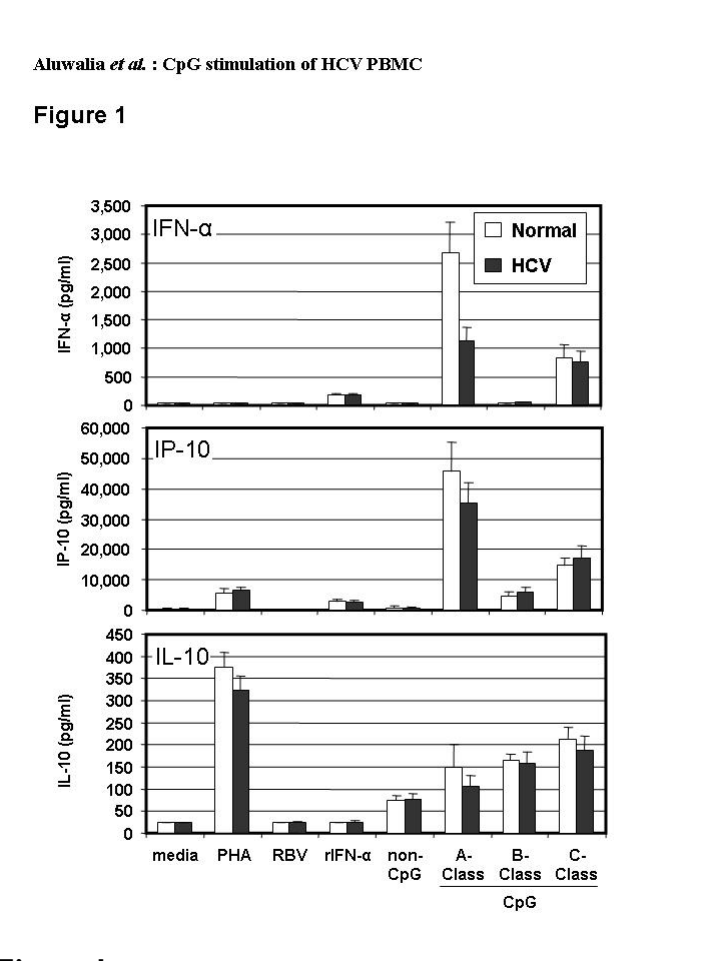
Levels of cytokines secreted by PBMC from healthy (n = 9 to 12) or HCV-infected (n = 13 to 15) donors after 48 hr culture with media, recombinant IFN-alpha (rIFN-α, 125 IU/ml), ribavirin (RBV, 5 μM), non-CpG control ODN, A-Class, B-Class or C-Class CpG (all ODN at 6 μg/ml). White bars (Healthy) and black bars (HCV), show mean values and standard error of the means for each group of subjects. The lowest limit of quantification for each of the parameters was as follows: IFN-α, 31.2 pg/ml, IL-10, 23.4 pg/ml and IP-10, 7.8 pg/ml.

Healthy donor PBMC secreted the highest levels of IFN-α and IP-10 (Figure 1). Consistent with a previous report, secretion was greatest with A-Class, less with C-Class, and least with B-Class CpG [38]. HCV PBMC yielded results similar to that of healthy PBMC for B- and C-Classes but produced significantly less IFN-α (p = 0.02) and a trend to less IP-10 with A-Class CpG. All CpG classes induced similar IL-10 levels in healthy and HCV PBMC (Figure 1).

**Figure 2:**
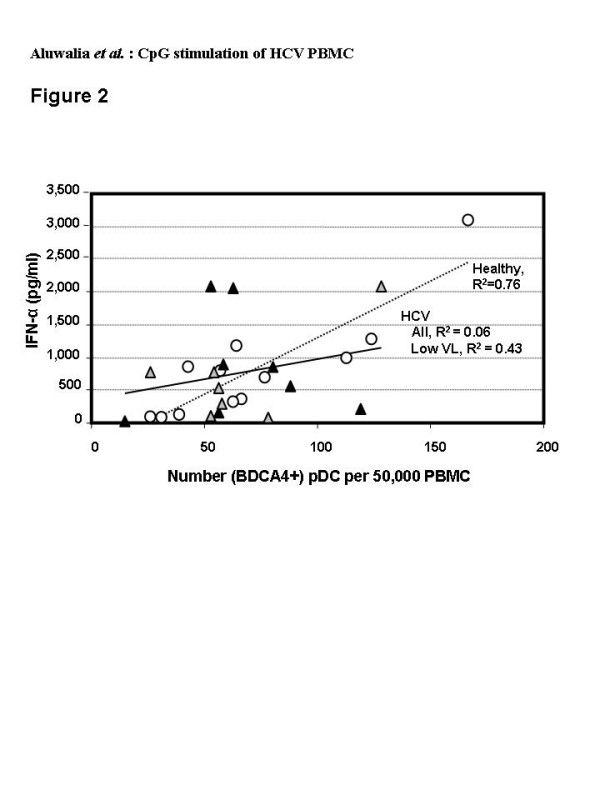
Flow cytometric analysis of pDC in freshly isolated PBMC from healthy (open circles, n = 12) and HCV-infected (grey or black triangles, n = 15) donors; HCV donors with low viral load at baseline (< 600,000 IU/ml) are indicated by grey triangles. Numbers of pDC counted among 50,000 events by flow cytometry of lineage negative, CD11c negative, HLA-DR+, BDCA4+ cells are plotted against the amount of IFN-α secreted by 1× 106 cells cultured for 48 hrs in the presence of the C-Class CpG at 6 μg/ml. Each point represents the results for an individual subject (average of duplicate assays).

**Figure 3:**
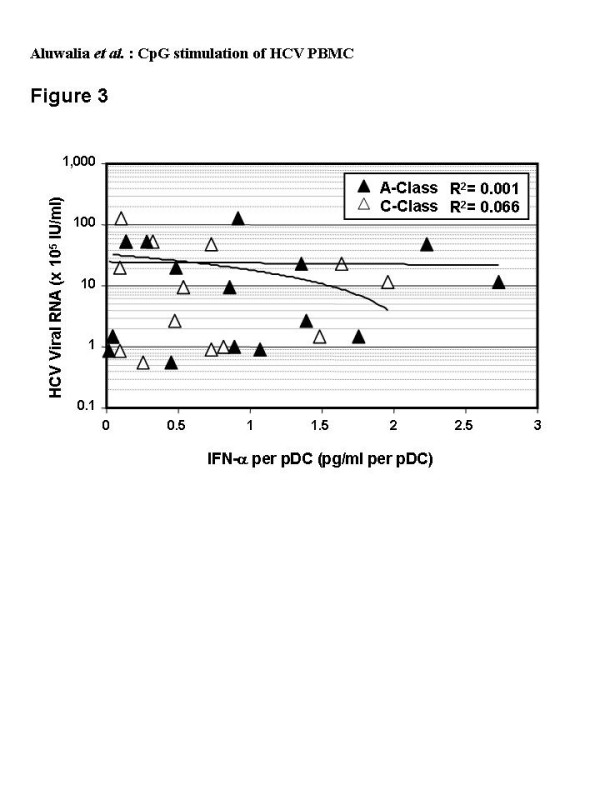
Correlation of blood levels of HCV RNA and levels of IFN-α secreted per pDC from individual HCV-infected donors (n = 15). The amount of IFN-α secreted by 1 × 10^6 ^cells cultured for 48 hrs with A-Class (black symbols) or C-Class (white symbols) CpG (6 μg/ml) was divided by the number of pDC (lineage negative, CD11c negative, HLA-DR+ and BDCA4+), counted among 50,000 events by flow cytometry and plotted against HCV RNA levels for the same subjects.

Two methods were used to quantify pDC in CD11c negative, HLA-DR positive cells: (i) BDCA-4 detection, which is specific to pDC but may be down-regulated upon pDC activation leading to concerns regarding undercounting, and (ii) CD123 detection, which is also expressed on basophils [46] [N.B. basophils are negative for HLA-DR]. Both methods yielded similar numbers of pDC from healthy (73 ± 42 and 56 ± 27 respectively, mean ± SD of 50,000 events) and HCV-infected (66 ± 30 and 58 ± 23) donors. Linear regression demonstrated a good correlation between number of pDC (BDCA-4 analysis) and amount of IFN-α secreted in response to C-Class CpG for normal donors (R^2 ^= 0.76). This was not identified in HCV donors (R^2 ^= 0.06) although a better correlation (R^2 ^= 0.43) was observed for HCV subjects with low blood levels of HCV RNA (< 600,000 IU/ml) (Figure 2). Similarly, A- and B-Class CpG stimulated IFN-α secretion that was well correlated with the number of pDC in normal (R^2 ^= 0.50 or 0.51 respectively) but not HCV (R^2 ^= 0.04 or 0.09) PBMC (not shown). The amount of IFN-α produced per pDC varied widely with HCV PBMC and did not correlate with viral RNA blood levels (Figure 3).

### PBMC proliferation

**Figure 4:**
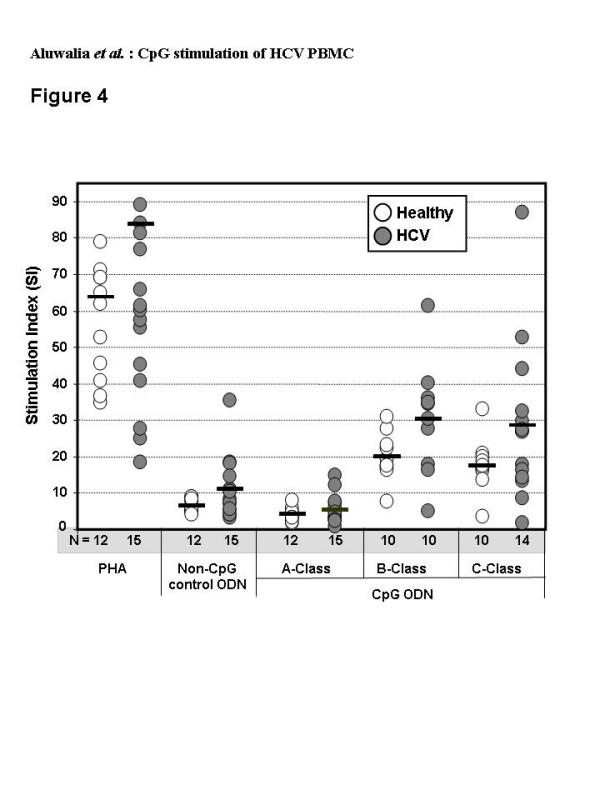
Proliferative responses in PBMC from healthy (open circles, n = 10 to 12) or HCV-infected (filled circles, n = 10 to 15) donors after incubation with A-, B- or C-Class CpG (6 μg/ml), positive control PHA (10 μg/ml) or non-CpG control ODN (6 μg/ml) for 5 days, then pulsing with 3H-thymidine for 16 to 18 hours. Horizontal bars represent the group means for stimulation indices (SI = cpm with PHA or ODN/cpm with media alone).

Under the culture conditions used, CpG-induced PBMC proliferation is thought to be mostly B cell related [47]. As previously reported [38], proliferation of PBMC from healthy donors was weak with A-Class but strong with B- and C-Class CpG. B- and C-Classes had similar effects at high concentration (~1 μM) (Figure 4) but at low concentration (~0.5 μM) the B-Class was more potent (p < 0.03, not shown). The non-CpG control ODN caused some proliferation, which is attributed to the weak TLR9-dependent stimulation of cells by the phosphorothioate backbone [48]. This was greater than that seen with the A-Class chimeric backbone (p = 0.0023). There were no significant differences in the proliferative responses between PBMC from healthy and HCV-infected subjects with any of the three classes of CpG (p > 0.05).

### Effects of IntronA and ribavirin

As expected, IP-10 was induced by IntronA (Figure 1). The amount was similar to that with B-Class but significantly less than with A- or C-Class CpG (p < 0.002). IntronA did not induce proliferative responses (data not shown) or IL-10 secretion (Figure 1).

**Figure 5:**
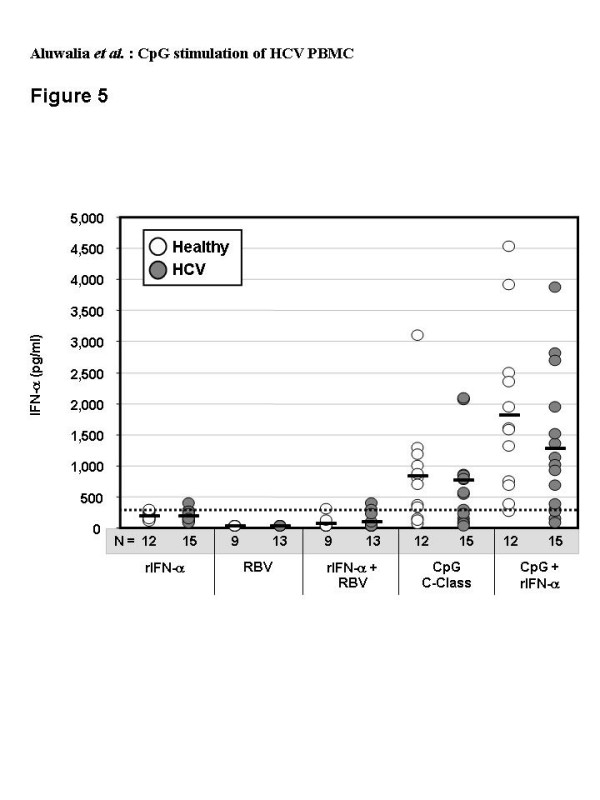
Levels of IFN-α secreted by PBMC from healthy (n = 9 to 12) or HCV-infected (n = 13 to 15) donors after 48 hr culture with recombinant IFN-α (rIFN-α, 125 IU/ml), ribavirin (RBV, 5 μM), rIFN-α plus ribavirin, C-Class CpG (6 μg/ml) or CpG plus rIFN-α. Horizontal black bars show group mean values, and numbers of subjects (n) in each group are indicated below the X-axis. The background level of IFN-α deemed to be contributed by the added rIFN-α alone, as measured in control B-cell line experiments (334 pg/ml), is shown by the hatched line.

The IFN-α ELISA assay does not differentiate between exogenous and endogenous forms. To determine whether IntronA induced IFN-α secretion from pDC we used EBV-immortalized B cell lines. These cells have IFN-α receptors but do not produce IFN-α which allows for the amount of IntronA remaining after 48 hr culture to be estimated. Seventeen experiments adding IntronA (125 IU/ml) to five different B-cell lines for 48 hr gave a mean level over media background of 172 ± 81 pg/ml. This was deemed to be a better estimate than measuring IFN-α after spiking media with IntronA (319 ± 112 pg/ml, n = 13) since metabolic degradation by cultured cells was expected. Amounts of IFN-α in supernatants of HCV or healthy PBMC and B-cell lines cultured with IntronA were similar (p < 0.05) indicating IntronA does not induce significant IFN-α secretion (Figure 5).

Ribavirin alone or in combination with IntronA did not induce significant IFN-α secretion (Figure 5). Ribavirin alone also failed to induce IP-10 or IL-10 secretion (Figure 1).

### IntronA combined with CpG

IntronA combined with C-Class CpG significantly augmented the amount of IFN-α secreted relative to CpG alone (p < 0.02) (Figure 5). Individual data revealed a greater than 50% increase over CpG alone for all donors tested.

**Figure 6:**
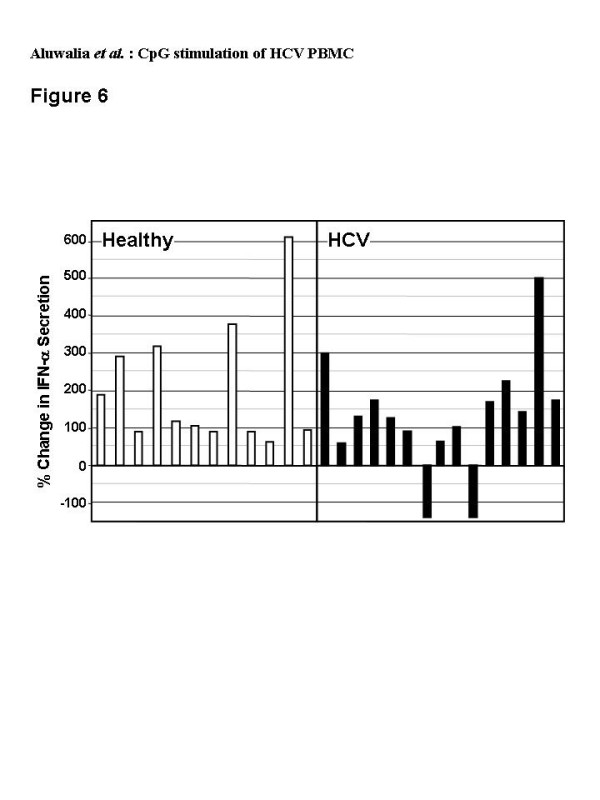
Percent change in amount of IFN-α secreted by PBMC after 48 hr culture with rIFN-α (125 IU/ml) plus C-Class CpG (6 μg/ml) over that with CpG alone. The amount of IFN-α measured for rIFN-α alone for each subject was subtracted from the rIFN-α plus CpG value to account for rIFN-α itself remaining in the culture media. Individual data is shown for PBMC from healthy (open bars, n = 12) or HCV-infected (closed bars, n = 15) donors.

**Figure 7:**
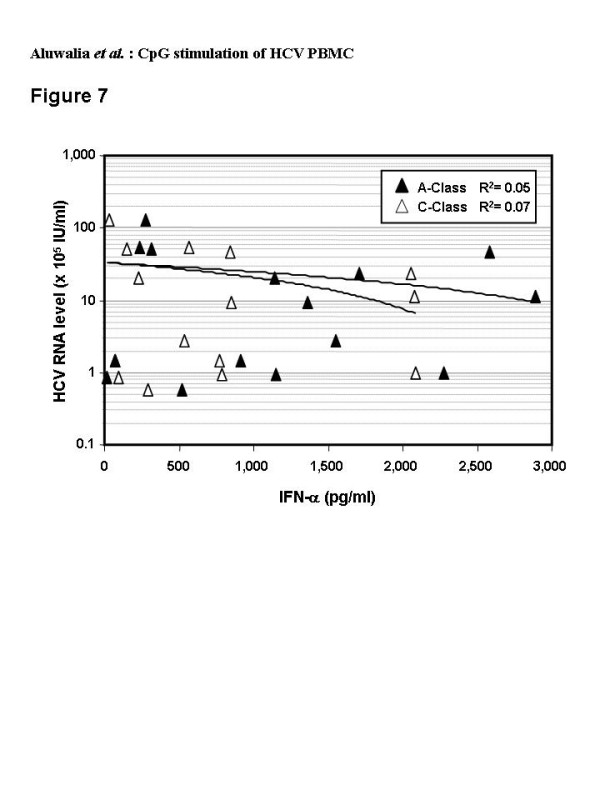
Correlation of blood levels of HCV RNA and levels of IFN-α secreted by PBMC from individual HCV-infected donors (n = 15) after 48 hr culture with A-Class (black symbols) or C-Class (white symbols) CpG (6 μg/ml). The 7 HCV subjects with low viral load (< 600,000 IU/ml) had R2 values of 0.07 and 0.005 for the A- and C-Class CpG respectively.

All (12/12) healthy and most (13/15) HCV donors achieved a minimum 50% increase and 4/12 and 3/15 produced a minimum 100% increase in IFN-α secretion over CpG alone with addition of Intron A (Figure 6). Synergy did not correlate with HCV genotype or viral RNA level (R^2 ^< 0.2) (Figure 7); both of these viral characteristics influence therapeutic response [18,49,50].

No augmentation was seen for CpG-induced IP-10 or IL-10 or PBMC proliferation (data not shown). It is possible that CpG alone induced maximal IP-10 and hence no additive effects were noted despite higher levels of IFN-α. A similar phenomenon with IFN-α and IP-10 induction by B-Class CpG has been described [51].

## Discussion

Recognition of the need to overcome immune dysfunction in chronic HCV and induce strong virus-specific T cell responses has led to the evaluation of immune modulators alone and in combination with current HCV therapies. We demonstrated CpG-induced PBMC stimulation in both healthy and HCV-infected donors. Of note, high level IFN-α secretion by pDC was produced following A- and C-Class CpG induction [32],

A-Class CpG induce very high amounts of IFN-α secretion from pDC [37,52]. We found diminished IFN-α levels with chronic HCV compared to healthy donor PBMC. This is consistent with a number of earlier studies of A-Class CpG on PBMC [53-55] or purified pDC [56]. One study with purified pDC study failed to reveal a difference [57]. In the present study, lower IFN-α secretion in those with HCV cannot be explained by reduced pDC numbers since IFN-α levels and pDC numbers did not correlate. One previous study identified similar levels of IFN-α in healthy and HCV pDC [54]. Reduced IFN-α secretion was attributed to reduced numbers of circulating pDC. In another study, levels of IFN-α per pDC were lower with HCV [55]. A greater than one hundred-fold reduced capacity in IFN-α production was attributed to immature phenotype and compartmentalization of pDC in the inflamed liver [55]. It is note worthy that in our evaluation PBMC stimulation with B- or C-Class CpG produced no differences in proliferation and cytokine secretion between health volunteers and HCV-infected study participants.

Structural differences between A- and C-Class CpG may account for variable IFN-α secretion outcomes. Monomeric molecules such as B-Class CpG can activate TLR9 but only the A- and C-Classes that form higher ordered structures can induce high levels of IFN-α secretion from pDC. This may be a consequence of cross-linkage with TLR9 receptors [38]. A-Class CpGs form large polymeric structures due to their poly-G regions whereas C-Class only form dimers. It has been proposed that pDC revert to a more immature state with chronic HCV infection [55] or that direct HCV infection of pDC may alter their ability to take up and/or respond to the larger A-Class structures [58].

HCV chronic carriers have dysfunctional pDC with impaired capacity to stimulate allogeneic T cells. This may be mediated by altered MHC expression and cytokine production that facilitate regulatory T cells development [56,59-61]. Reduced IFN-α secretion has been noted in response to a non-specific stimulus such as the herpes simplex virus [6] and poly(I:C), a TLR3 ligand [53]. As such, the ability of both A- and C-Class CpG to induce IFN-α secretion in PBMC from HCV chronic carriers is notable. IFN-α secretion with C-Class CpG stimulation were similar between healthy donors and HCV infected participants but levels were more variable in the latter group. As a consequence, there was good correlation between the amount of IFN-α secreted and number of pDC in the sample for healthy PBMC but not in the HCV population.

A- and C-Class CpG produced similar levels and types of immune activation with the exception of B-cell proliferation which is more robust following C-Class stimulation. Both were more potent than B-Class. Based on these results, and earlier findings of stronger TLR9-dependent NFkB signaling with C-Class [38], a C-Class CpG (CPG 10101) was chosen for clinical testing in HCV in combination with PEG-IFN-α and/or ribavirin. Thus, our evaluation of the interactions between these medications and CpG in HCV-infected donor PBMC stimulation tests is relevant. As observed in other studies [25], IntronA and/or ribavirin had limited effect on the immune parameters tested. This suggests that these medications may be suboptimal for inducing T cell responses thought to prevent virological relapse following HCV antiviral therapy. Combining IntronA with C-Class CpG augmented IFN-α secretion from pDC. This is consistent with other work suggesting that pre-treatment of human PBMC with recombinant IFN-α primes pDC to respond to the stimulatory effects of bacterial DNA [62]. Even thought no ribavirin-CpG synergy was detected in the present *in vitro *study, such synergy might be realized *in vivo*. *In vivo *evaluation has demonstrated that ribavirin may diminish Th2 cytokines including IL-10 thereby enhancing Th1 responses [21] and reducing regulatory T cell induction [63]. Blocking the IL-10 receptor on HCV PBMC results in increased HCV-specific IFN-γ producing T cells [23]. Hence, ribavirin might enhance *in vivo *responses to CpG by perturbing IL-10 activity.

## Conclusion

In summary, the C-Class of CpG molecules possess effective immunostimulatory effects on PBMC from chronic HCV donors and might provide complementary and additional mechanisms of action to current HCV therapies.

## Authors' contributions

CC participated in study design, was responsible for study recruitment and manuscript preparation, NA, SE and JV conducted the analysis and contributed to the manuscript preparation, AK and HD conceived of the study, participated in study design and manuscript preparation. All authors have read and approved the final manuscript.
